# Unravelling the Interplay Between *Vairimorpha ceranae* and Viruses in Geographically Isolated Honey Bee (Apis mellifera) Populations

**DOI:** 10.1111/eva.70294

**Published:** 2026-07-15

**Authors:** Ana R. Lopes, Matthew Low, Raquel Martín‐Hernández, Mariano Higes, Joachim R. de Miranda, M. Alice Pinto

**Affiliations:** ^1^ CIMO, LA SusTEC Instituto Politécnico de Bragança Bragança Portugal; ^2^ Department of Ecology Swedish University of Agricultural Sciences Uppsala Sweden; ^3^ Centro de Investigación Apícola y Agroambiental (CIAPA), IRIAF Instituto Regional de Investigación y Desarrollo Agroalimentario y Forestal Marchamalo Spain

**Keywords:** Azores, honey bee, interaction, synergy, *Vairimorpha ceranae*, *Varroa destructor*, viruses

## Abstract

Viral infections and the microsporidian *Vairimorpha ceranae* (‘vairimorpha’) have been involved in elevated honey bee ( *Apis mellifera* ) colony losses as a result of their individual or combined actions, with the ectoparasitic mite *Varroa destructor* (‘varroa’) often exacerbating these effects. This study investigates the relationship between vairimorpha and several common honey bee viruses within the unique ecological context of the remote mid‐Atlantic Azores archipelago, which comprises three main islands where varroa is present and six islands where varroa is still absent. We sampled 494 colonies across these islands and employed RT‐qPCR to detect and quantify vairimorpha and nine common honey bee viruses, of which seven were detected. The data were modelled using a Bayesian approach, which estimated the likelihood of an effect of the presence of vairimorpha on the loads of each of the viruses on varroa‐infested and varroa*‐*free islands. Both vairimorpha and the seven bee viruses were differentially distributed among the islands. An additive relationship was found between vairimorpha and both black queen cell virus (BQCV) and Lake Sinai virus (LSV), the latter driven almost exclusively by one strain (LSV‐2), and this relationship was stronger on the varroa‐infested islands (Pr = 96.2%) than on the varroa‐free islands (Pr = 69.1%). No association was found between vairimorpha and the two main strains of DWV (DWV‐A and DWV‐B) across all islands, although an antagonistic relationship may exist between vairimorpha and the rare DWV‐C strain, found only on varroa‐free islands. This study supports previous findings and shows the importance of possible additive relationships among co‐infecting honey bee pathogens and parasites.

## 1 Introduction

The ectoparasitic mite *Varroa destructor* (hereafter ‘varroa’) and the microsporidian *Vairimorpha ceranae* (Bojko et al. 2025) (hereafter ‘vairimorpha’) are among the most detrimental biotic stressors of honey bee ( *Apis mellifera* ) colonies and the causative agents of high annual losses worldwide (Higes et al. 2008; Traynor et al. 2020; Hristov et al. 2021). They both have ancestral roots in Asia and have rapidly adapted to *A. mellifera* after a host shift in the 20th century from their original host, *Apis cerana* (Anderson and Trueman 2000; Higes et al. 2006). While varroa directly harms *A. mellifera* by feeding on the fat body and haemolymph of both pupae and adults (Ramsey et al. 2019; Han et al. 2024), the most important damage is caused by the transmission of detrimental viruses, primarily Deformed wing virus (DWV; *Iflavirus aladeformis*) or members of the AKI‐virus complex: Acute bee paralysis virus (*Aparavirus apisacutum*), Kashmir bee virus (*Aparavirus kashmirense*) and Israeli acute bee paralysis virus (*Aparavirus israelense*) (Traynor et al. 2020; Lopes et al. 2024b). Varroa can also have an indirect detrimental effect on parasitized honey bees by facilitating the development of opportunistic secondary viruses that are not vectored directly by varroa, such as Sacbrood virus (SBV; *Iflavirus sacbroodi*), Black queen cell virus (BQCV; *Triatovirus nigereginacellulae*) and Chronic bee paralysis virus (CBPV; *unassigned*), but instead exploit the weakening of the host immune defenses by varroa and its primary viruses (Chantawannakul et al. 2006; Traynor et al. 2020). Vairimorpha infects the epithelial cells of the honey bee midgut, disrupting the normal absorption of nutrients and digestion processes (Higes et al. 2020), which leads to nutritional stress and decreased energy levels in infected bees (Higes et al. 2007). Moreover, the injury caused to the intestinal lining may also enable the infiltration of viral particles into the haemolymph, exacerbating viral infections (Martin et al. 2013; Martín‐Hernández et al. 2018). Vairimorpha infection can also affect honey bees by reducing their immunocompetence, which may again facilitate the opportunistic development of viral infections (Antúnez et al. 2009; Higes et al. 2013; Martín‐Hernández et al. 2018).

The worldwide distribution of varroa and vairimorpha means that there is a high chance that both parasites co‐occur in a colony, which in turn creates the opportunity for synergistic, antagonistic, or additive interactions between varroa, vairimorpha and a range of common pathogenic bee viruses(Martín‐Hernández et al. 2018). Several studies have addressed vairimorpha‐virus interactions, and these have consistently found synergies between vairimorpha and BQCV, CBPV, or Lake Sinai virus strain 2 (LSV‐2) (Bailey et al. 1983; Dainat et al. 2012; Toplak et al. 2013; Doublet, Labarussias, et al. 2015; Traynor et al. 2016; Gajda et al. 2021). However, when it comes to the interaction between vairimorpha and DWV, both synergistic, antagonistic, and even no interaction has been reported (Costa et al. 2011; Martin et al. 2013; Doublet, Natsopoulou, et al. 2015; Little et al. 2016; Bordin et al. 2022). All these studies were conducted in locations where varroa had already been long established and therefore might already have affected the original relationships between vairimorpha and these viruses by the time the studies were conducted. To elucidate the secondary effect of long‐term varroa infestation on vairimorpha‐virus interactions it is useful to have access to a good number of clearly delineated and relatively proximate regions evenly divided between regions with and without a particular biological stressor (such as varroa and vairimorpha): effectively a clean, binary experimental design at scale. The Azores mid‐Atlantic island archipelago is one such place, as it comprises islands where both varroa and vairimorpha are either categorically present or absent (Ferreira et al. 2020; Lopes et al. 2022). Varroa successfully invaded three of the nine Azorean islands in the early 21st century, while the remaining six islands are still varroa‐free, a status that is maintained through continuous official surveillance programmes, including regular apiary inspections and active screening for *Varroa destructor* (European Commission, 2019). Vairimorpha is more widely spread, with only two islands (Flores and Santa Maria) naïve to this pathogen (Lopes et al. 2022). Herein, we capitalised on this unique natural experimental framework of varroa‐infested and varroa‐free islands to investigate how the long‐term mite presence influences the associations between vairimorpha and a range of honey bee viruses and their major strains. Assuming that both varroa and vairimorpha are by nature detrimental to colony health, triggering opportunistic virus infections (in addition to the varroa‐vectored viruses), we hypothesised that colonies with vairimorpha would exhibit higher viral loads than those without, and that effect would be stronger on varroa‐infested than on varroa‐free islands.

## 2 Materials and Methods

A total of 494 colonies of *Apis mellifera iberiensis* were sampled across the Azores archipelago (Ferreira et al. 2020). Sampled colonies were housed in movable‐frame hives and managed conventionally; on varroa‐infested islands, colonies were treated at least once a year with approved varroacides, in accordance with national regulations. Sample collection occurred on two broad occasions: first during July and August of 2014 and 2015 (one pooled occasion), and then again 5 years later, during July and August of 2020 as described in Lopes et al. 2024b and Table S1, with the final sample sizes for all islands and both sampling occasions provided in Table S2. These colonies were screened by PCR for vairimorpha and nine viruses, including DWV, the AKI complex viruses, BeeMLV, CBPV, BQCV, LSV, and SBV. To that end, DNA and RNA were extracted from pools of 30 worker bees (diploid sterile females) per colony using NucleoSpin Tissue commercial kit (Macherey‐NagelTM, Düren, Germany) and Monarch Total RNA Miniprep kit (NewEngland Biolabs Inc., MA, USA), respectively, following the manufacturer's instructions. RNA was subsequently converted to cDNA using Bio‐Rad iScriptTM cDNA Synthesis Kit. The DNA templates were subjected to PCR in QuantuStudio 5 apparatus (Applied Biosystems, MA, USA) using SyberGreen chemistry (2X iTaq Universal SYBR Green Supermix, Biorad) to establish vairimorpha infection status of each colony (Lopes et al. 2022), while the cDNA templates were analysed by qPCR to detect and quantify the levels of nine RNA viruses (Lopes et al. 2022; Lopes et al. 2024a; Lopes et al. 2024b). The DNA protocol is described in Lopes et al. (2022) whereas RNA extraction protocols, cDNA synthesis, and qPCR reactions are described in Lopes et al. (2024b). For both vairimorpha infection status and virus detection, samples were classified as positive for the particular target amplified if they passed the amplification cycle threshold set by the final dilution of the seven‑step 10‑fold serial dilution standard curve. The absolute quantification of each virus was performed using a duplicate standard curve of a seven step 10‑fold serial dilution series of a synthetic positive control of known absolute concentration, ranging from 10^1^ to 10^7^ target copies per μL of template. The loads were converted to target copies per bee by multiplying the estimated copy number per PCR reaction with the various dilution factors incurred during processing, extraction, cDNA synthesis and qPCR. These PCR (vairimorpha) and qPCR (viruses) assays produced binary data for the presence/absence of vairimorpha in each colony, and continuously distributed data for the amounts of each virus in a typical individual bee from each colony. These, together with the binary varroa status of each island, are the main variables available for formal analyses. Because viral amplification is largely exponential, virus load tends to follow a log‐linear distribution (de Miranda et al. 2013), and was therefore log_10_‐transformed during analysis in order to normalize the data, resulting in a LogNormal model structure. A generalised linear mixed model (GLMM) was used to assess the effect of categorical colony‐level vairimorpha infection status on the loads of each virus. The model was structured relating viral load to vairimorpha colony status and sampling occasion, which were included as the fixed effects, while apiary ID was included as a random effect in the intercept to account for variation among apiaries. The formal model description is presented as follows:

**Observation model 1:**

$$y_{i}\sim N\left(\mu_{i},\sigma \right)$$

$$\mu_{i}=\alpha_{j\left[i\right]}+\beta_{1}\times {\text{ceranae}}_{i}+\beta_{2}\times {\text{year}}_{i}$$

$y_{i}$ denotes virus loads for observation *i*.

$\alpha_{j\left[i\right]}$ is the intercept for the group *j* corresponding to the observation *i*.

${\text{ceranae}}_{i}$ represents the presence/absence of vairimorpha infection.

${\text{year}}_{i}$ is the sampling occasion.

**Random effects (hierarchical structure)**.

Group‐level (apiary) intercepts were modelled as: $\alpha_{j}\sim N\left(\mu_{\alpha },\sigma_{\alpha }\right)$

**Priors (weakly informative):**


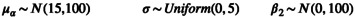




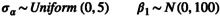

Separate models were run for colonies from varroa‐infested (Vd+) and varroa‐free (Vd‐) islands. A further refinement of this approach was employed by also considering the different major dominant strains of DWV (DWV‐A, DWV‐B and DWV‐C) and LSV (LSV‐2, LSV‐3 and LSV‐9) previously identified for each sample using a meta‐barcoded Illumina amplicon sequencing approach. Briefly, amplicon sequencing libraries were constructed for all DWV‐, LSV‑, CBPV‑, and SBV‑positive samples plus a representative subset of 174 BQCV‑positive samples. Metabarcoding sequencing libraries were prepared using a two‑stage PCR strategy and sequenced on an Illumina MiSeq platform using 2 × 250 bp chemistry. The sequencing reads were demultiplexed by their unique barcodes, characterised genetically in Galaxy using Mothur, and filtered, counted and pooled to produce a quantitative ASV read‐count file for each virus and virus strain, which is the data used in the current study (see details in Lopes et al. 2024a; Lopes et al. 2024b). To further investigate the relationship between vairimorpha, varroa, and viral loads, we implemented a more complex modelling framework designed to capture the impact of potential interaction effects between varroa and vairimorpha on the quantitative viral dynamics. However, due to the distributional properties and hierarchical structure of the DWV, SBV, and CBPV datasets, model convergence could not be achieved for these viruses. Alternative model specifications using more informative priors were explored (Table S3), but these did not lead to reductions in parameter uncertainty or more reliable results. This likely reflects the limited amount of information in the data for these virus–vairimorpha combinations. Consequently, our interaction analyses were restricted to LSV and BQCV, the only viral datasets for which the models exhibited stable and biologically interpretable convergence. The model was structured relating viral load to vairimorpha colony status, varroa island status, and sampling occasion, which were included as the fixed effects, while apiary ID was included as a random effect in the intercept to account for variation among apiaries. The formal model description is presented as follows:

**Observation model 2:**

$$y_{i}\sim N\left(\mu_{i},\sigma \right)$$

$$\begin{array}{c} \mu_{i}=\alpha_{j\left[i\right]}+\beta_{1}\times {\text{ceranae}}_{i}+\beta_{2}\times {\text{year}}_{i}+\beta_{3}\times {\text{mite}}_{i}\\ \;+\beta_{4}\left({\text{ceranae}}_{i}\times {\text{mite}}_{i}\right) \end{array}$$

$y_{i}$ denotes virus loads for observation *i*.

$\alpha_{j\left[i\right]}$ is the intercept for the group *j* corresponding to the observation *i*.

${\text{ceranae}}_{i}$ represents the presence/absence of vairimorpha infection.

${\text{year}}_{i}$ is the sampling occasion.

${\text{mite}}_{i}$ represents the presence/absence of varroa.

**Random effects (hierarchical structure)**.

Group‐level (apiary) intercepts were modelled as: $\alpha_{j}\sim N\left(\mu_{\alpha },\sigma_{\alpha }\right)$

**Priors (weakly informative):**

$$\mu_{\alpha }\sim N\left(15,100\right)\;\;\;\sigma \sim \text{Uniform}\left(0,2\right)\;\;\;\beta_{2}\sim N\left(0,100\right)$$

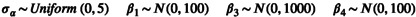

The modelling was implemented using a Bayesian framework assisted by JAGS in R (Plummer 2003), utilising minimally informative priors. The Markov chain Monte Carlo chains underwent sampling for 10,000 iterations of post‐convergence confirmation through visual inspection of stability. Posterior predictive checks were conducted to evaluate the model fit. A Bayesian framework was chosen because it allows effect sizes to be quantified in probabilistic terms, including both their magnitude and direction. For interpretation, the posterior probability of the effect size was classified as follows: > 95% indicating strong evidence for an effect, > 90% indicating moderate evidence, 70%–90% indicating weak‐to‐moderate evidence, and < 70% indicating no evidence. All analyses were conducted using the R software version 4.5.1 (R Core Team 2022).

## 3 Results

Viruses of the AKI complex and BeeMLV were not detected in any of the colonies. The 26 colonies positive for CBPV were all negative for vairimorpha and could thus not be included in any structured analysis with reference to the absence or presence of vairimorpha (Lopes et al. 2024a). This meant that only BQCV, SBV, DWV, and LSV could be analysed formally in relation to the presence or absence of vairimorpha.

**FIGURE 1:**
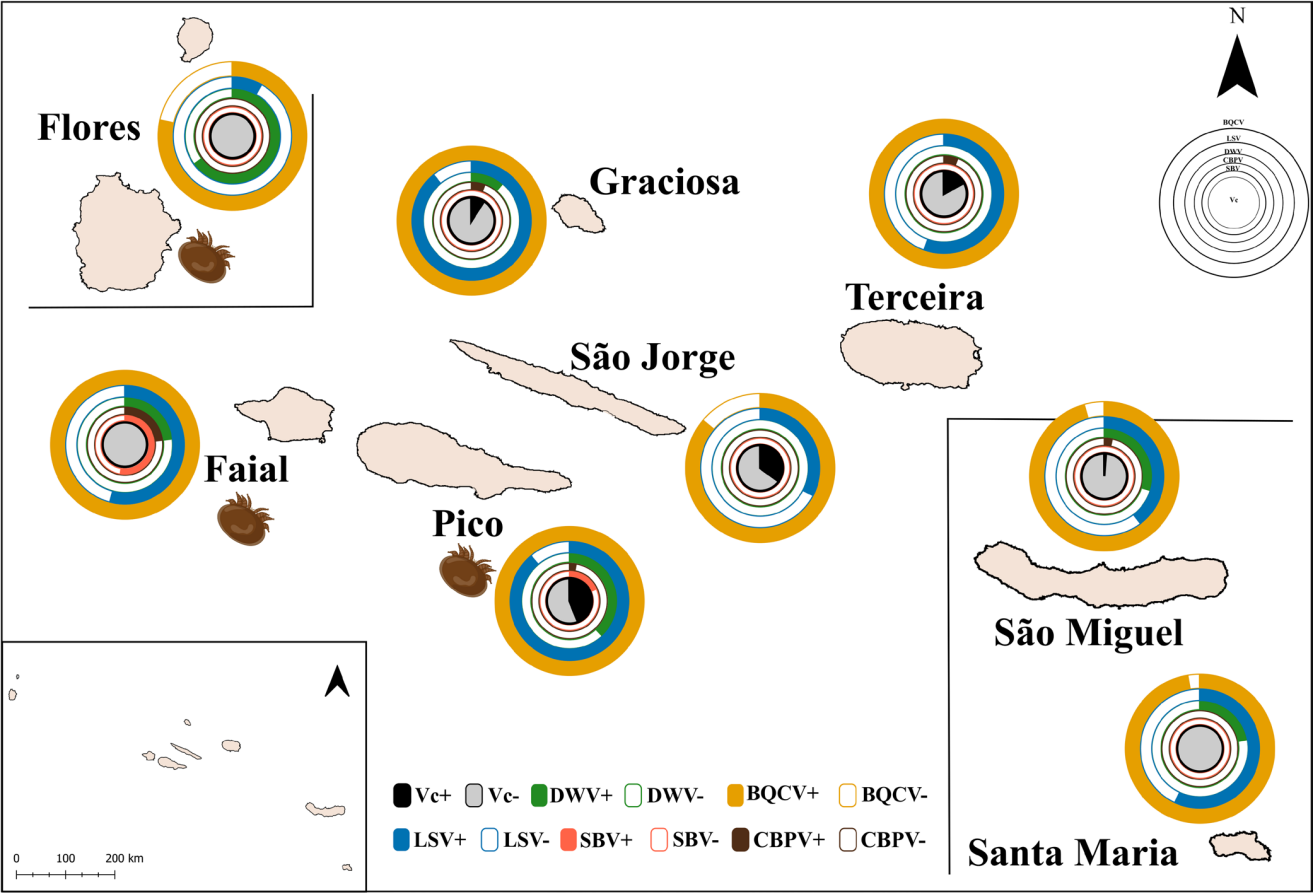
Prevalence of *Vairimorpha ceranae* and viruses in honey bee colonies across the eight Azorean islands. The outer rings represent the prevalence of viruses and the inner pie chart the prevalence of *V. ceranae*. Varroa mite icon indicates the varroa‐infested islands.

BQCV and LSV were present on every single island, contrasting with SBV, which occurred only on two varroa‐infested islands (Pico and Faial), and DWV, which was absent on two varroa‐free islands (Terceira and São Jorge) (Figure 1). Analyses of the DWV and LSV at the strain level provided a more detailed understanding of the epidemiology and evolution of bee viruses in the Azores. While the varroa‐infested islands were dominated by DWV‐A, DWV‐B, and LSV‐2, the varroa‐free islands were dominated by DWV‐C, LSV‐3, and the newly discovered LSV‐9 (Lopes et al. 2024a, 2024b). The single exception was São Jorge, which is varroa‐free but has a dominance of LSV‐2, that is, similar to the varroa‐infested islands.

**FIGURE 2:**
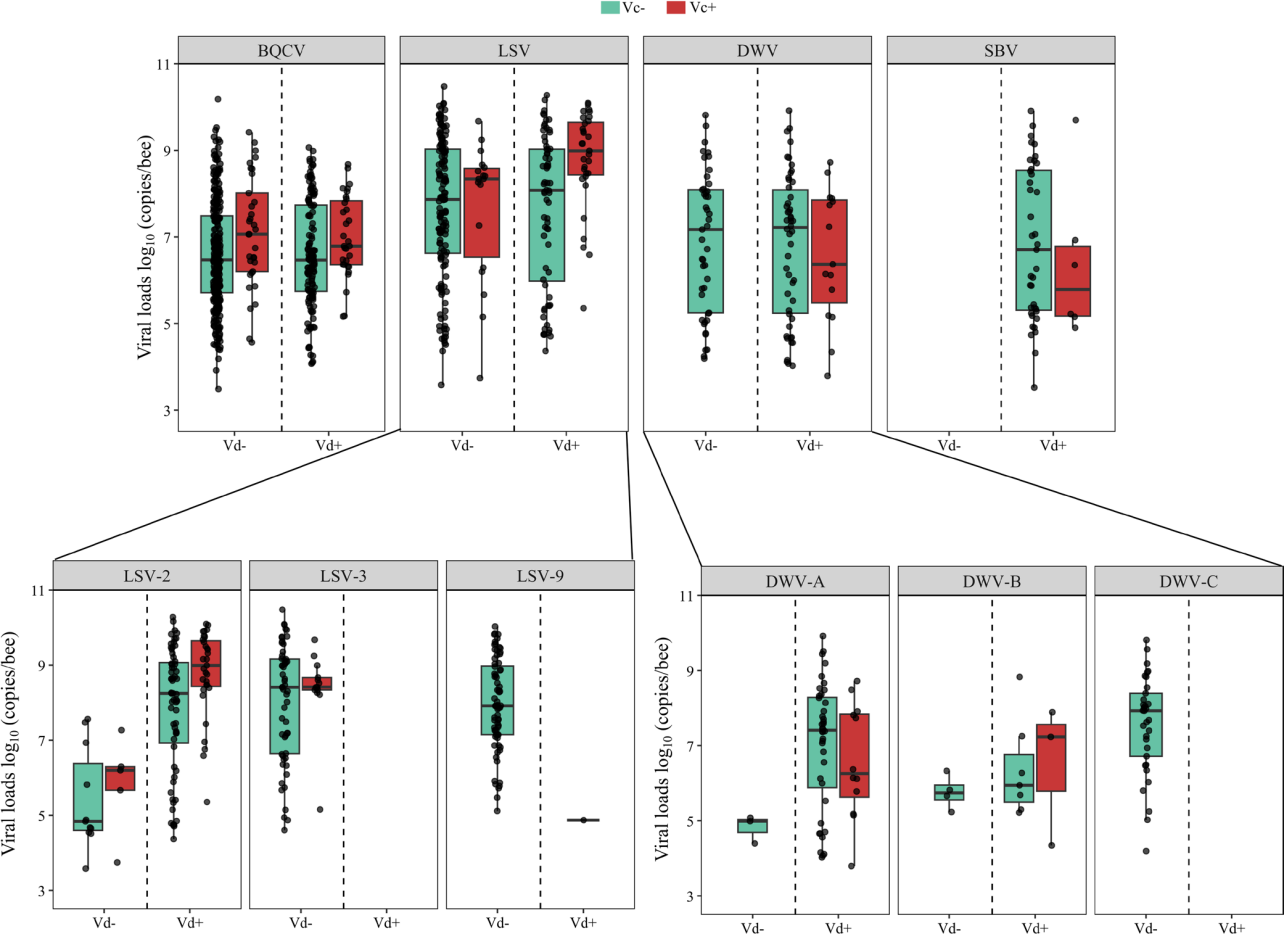
Viral loads for vairimorpha‐positive colonies (Vc+: Red boxplots) versus vairimorpha‐negative colonies (Vc‐: Green boxplots) for varroa‐infested (Vd+) and varroa‐free islands (Vd−) in the Azores. BQCV, Black queen cell virus; LSV, Lake Sinai virus; DWV, Deformed wing virus; SBV, Sacbrood virus. Viral loads are shown at the strain level for the multi‐strain viruses LSV and DWV.

Figure 2 compares the BQCV, LSV, DWV and SBV loads in vairimorpha‐positive colonies (Vc+) and vairimorpha‐negative colonies (Vc‐) according to the varroa status of the islands where these colonies were located, as well as a detailed breakdown of these loads by major strain for LSV and DWV. Notably, all colonies from the easternmost islands, Santa Maria and São Miguel, carrying the dominant variants DWV‐C and LSV‐9, as well as all colonies from Graciosa carrying DWV‐A, all of which are varroa‐free tested negative for vairimorpha. In contrast, BQCV and the other variants of LSV and DWV were detected in both Vc + and Vc‐ colonies across the Azores. The median loads of BQCV and LSV were higher in Vc + colonies (6.96 log_10_ copies/bee; IQR = 1.54 for BQCV and 8.61 log_10_ copies/bee; IQR = 1.20 for LSV) than in Vc‐ colonies (6.47 log_10_ copies/bee; IQR = 1.88 for BQCV and 7.92 log_10_ copies/bee; IQR = 2.51 for LSV). The opposite pattern was observed for DWV and SBV, with Vc + colonies having lower DWV and SBV loads (6.37 log_10_ copies/bee; IQR = 2.37 for DWV and 5.78 log_10_ copies/bee; IQR = 1.61 for SBV) than Vc‐ colonies (7.18 log_10_ copies/bee; IQR = 2.86 for DWV and 6.70 log_10_ copies/bee; IQR = 3.23 for SBV). Bayesian modelling confirmed that only BQCV and LSV were strongly influenced by the presence of vairimorpha, with a mean load increase of 0.59 ± 0.42 log_10_ copies/bee (Pr = 92.5%) and 1.72 ± 0.55 log_10_ copies/bee (Pr = 99.9%), respectively. SBV showed a weak‐to‐moderate negative effect, with a mean decrease of 1.64 ± 2.37 log_10_ copies/bee in Vc + colonies (Pr = 78.4%), whereas vairimorpha had no clear influence on DWV loads (Pr = 54.5%).

Table 1 shows the results of the Bayesian modelling aimed at assessing the effect of island varroa status on the impact of vairimorpha presence on viral loads in colonies across the Azores. On the varroa‐infested islands, the presence of vairimorpha had no effect on DWV (Pr = 55.7%), a weak‐to‐moderate negative effect on SBV (Pr = 80.4%, i.e., Pr = 19.6% for a positive effect), a weak‐to‐moderate positive effect on BQCV (Pr = 88.3%), and a strong positive effect on LSV (Pr = 96.1%), specifically due to LSV‐2 (Pr = 96.2%). On the varroa‐free islands, vairimorpha only had a weak‐to‐moderate positive effect on LSV loads (Pr = 84.0%), and no major effect on BQCV, DWV or SBV. The reduced overall effect of vairimorpha on LSV loads on the varroa‐free islands can be linked to the absence of an effect on LSV‐2 loads (Pr = 69.1%), which on the varroa‐infested islands was the virus strain most strongly linked to vairimorpha.

**TABLE 1:** Estimates of log_10_‐loads (copies/bee) by virus and viral strains for vairimorpha‐positive colonies (Vc+) and vairimorpha‐negative colonies (Vc−) on *V. destructor‐*infested (Vd+) and *V. destructor* ‐free (Vd−) islands.

Vd + islands					Vd‐ islands			
	Vc−	Vc+	Effect size	Pr	Vc−	Vc+	Effect size	Pr
BQCV	6.67 ± 0.35	6.99 ± 0.43	0.33 ± 0.30	88.3%	6.72 ± 0.14	6.93 ± 0.28	0.21 ± 0.30	73.8%
LSV	7.37 ± 0.47	8.09 ± 0.57	0.72 ± 0.41	96.1%	7.49 ± 0.20	7.99 ± 0.45	0.50 ± 0.50	84.0%
LSV‐2	7.40 ± 0.45	8.08 ± 0.54	0.68 ± 0.39	96.2%	5.09 ± 0.79	5.57 ± 1.13	0.48 ± 1.06	69.1%
LSV‐3	—	—	—	—	8.04 ± 0.42	8.23 ± 0.70	0.19 ± 0.90	56.6%
DWV	7.40 ± 0.54	7.47 ± 0.72	0.07 ± 0.59	55.7%	—	—	—	—
DWV‐A	7.57 ± 0.6	7.32 ± 0.81	−0.25 ± 0.63	37.3%	—	—	—	—
DWV‐B	6.53 ± 0.89	7.02 ± 1.53	0.48 ± 1.44	64.0%	—	—	—	—
SBV	6.38 ± 0.66	5.54 ± 1.09	−0.84 ± 0.98	19.6%	—	—	—	—

*Note:* The viral loads are the mean ± standard deviation of the posterior distributions generated by Bayesian hierarchical GLM models that account for apiary and sampling occasion effects. Vairimorpha effect size is the mean expected difference in log_10_‐load estimates between Vc + and Vc− colonies. Pr is the probability that this difference results in a higher viral load on Vc + colonies. LSV‑9 and DWV (on Vd− islands) are not included in the table because all colonies that tested positive for these viruses were Vc−, and therefore the corresponding analyses were not performed; LSV‐3 analysis was only conducted on Vd− islands; SBV analysis was only conducted for Vd + islands.

**FIGURE 3:**
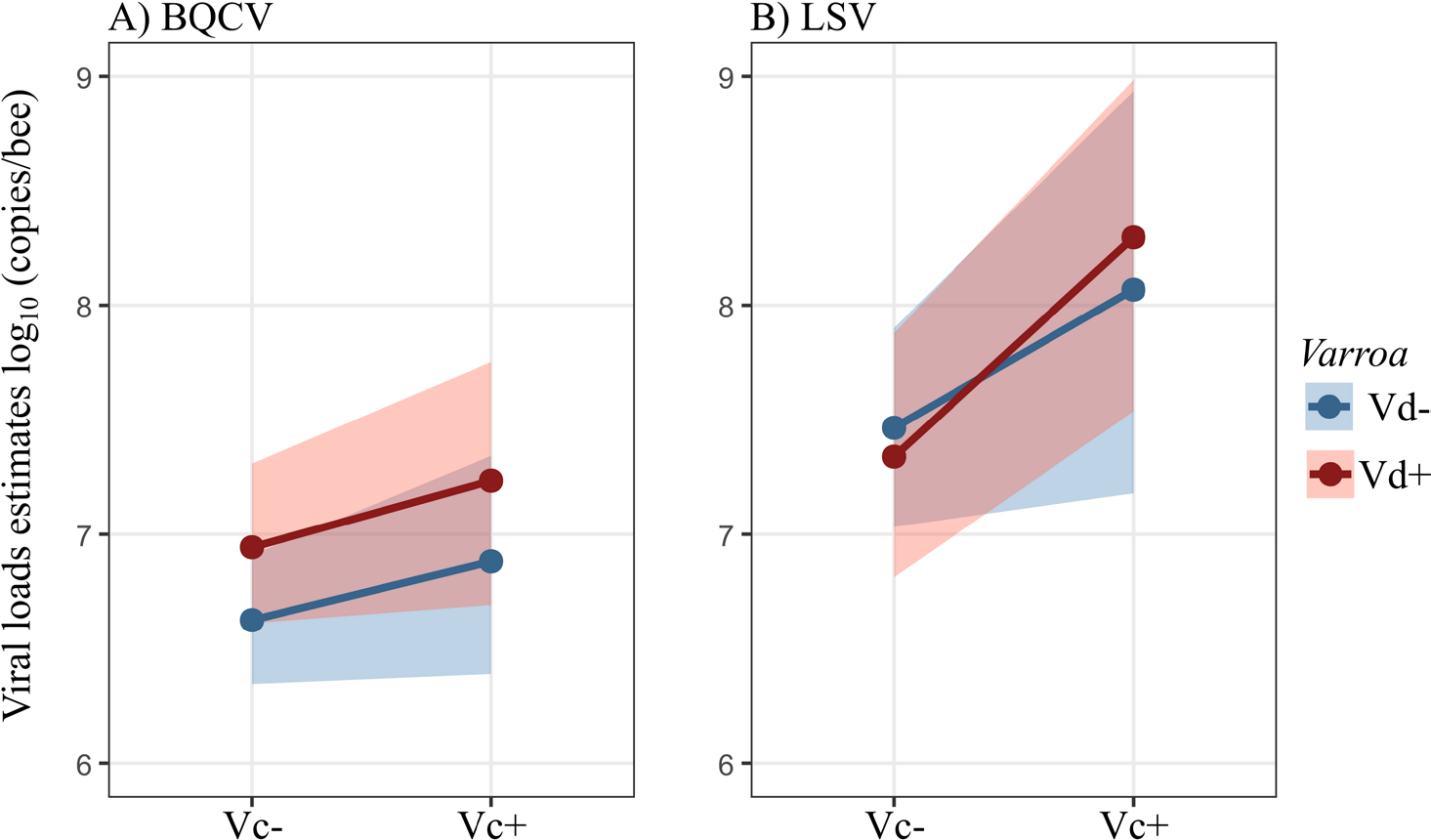
Effects of varroa and vairimorpha on viral loads in honey bee colonies. Predicted viral loads (log_10_ copies/bee) are shown for (A) BQCV and (B) LSV across vairimorpha‐positive (Vc+) and vairimorpha‐negative (Vc−) infection status. Lines and points indicate posterior mean estimates, and shaded areas represent 95% credible intervals. Colours distinguish varroa‑infested (red: Vd+) and varroa‐free (blue: Vd−) islands.

In line with these patterns, the interaction models further highlight that vairimorpha consistently contributes to elevated BQCV and LSV loads, particularly on varroa‐infested islands where both parasites co‐occur (Table 2). For BQCV, colonies exposed to both vairimorpha and varroa showed a markedly higher viral load (Pr = 98.8% for a positive combined effect), with positive effects estimated both for vairimorpha alone (Pr = 78.7%) and for varroa alone (Pr = 97.1%). This indicates that the presence of varroa tends to reinforce the already positive association between vairimorpha and BQCV (Figure 3A). For LSV, vairimorpha alone showed a weak‐to‐moderate positive effect on LSV load (Pr = 86.7%), whereas varroa alone had no effect (Pr = 30.6%). However, the combined effect of positive island varroa status and positive colony vairimorpha status had a strong increasing effect on LSV loads (Pr = 98.9%), demonstrating a clear synergistic association (Figure 3B). For BQCV, the impact of interaction effects between colony vairimorpha status and island varroa status are balanced and moderately supported (Pr > 0 = 80%–86%). In contrast, for LSV the effect of vairimorpha is strongly amplified in varroa presence (Pr > 0 = 99.4%; Table 2).

**TABLE 2:** Estimates log_10_‐loads (copies/bee) (mean log_10_ ± SD) and derived biological and interaction effects for BQCV and LSV under different co‐infection scenarios. Parameters A‐D concern the viral load estimates in the presence or absence of Varroa destructor (Vd) and Vairimorpha cerana (Vc). Parameters E‐G concern the biological effects of virus infection in the presence or absence of only Vairimorpha cerana, i.e., in the absence of Varroa destructor (E); of virus infection in the presence or absence of only Varroa destructor, i.e., in the absence of Vairimorpha cerana (F) and the biological effects of virus infection in the presence or absence of both varroa and vairimorpha in all combinations (G).

Virus	Category	Parameter	Mean (log_10_) ± SD	Effect size (log_10_) ± SD	Pr (> 0)
BQCV	Viral loads estimates	(A) Vd + and Vc+	7.25 ± 0.29		
		(B) Vd + and Vc−	6.95 ± 0.18		
		(C) Vd− and Vc+	6.88 ± 0.26		
		(D) Vd− and Vc−	6.63 ± 0.14		
	Biological effects	(E) vairimorpha only (C‐D)		0.25 ± 0.32	78.7%
		(F) varroa only (B‐D)		0.31 ± 0.18	97.1%
		(G) vairimorpha + varroa (A‐D)		0.62 ± 0.28	98.8%
	Interaction	Effect of vairimorpha in the presence of varroa (G‐F)		0.30 ± 0.28	81.2%
		Effect of varroa in the presence of vairimorpha (G‐E)		0.37 ± 0.43	85.5%
LSV	Viral loads estimates	(A) Vd + and Vc+	8.28 ± 0.37		
		(B) Vd + and Vc−	7.32 ± 0.27		
		(C) Vd− and Vc+	8.08 ± 0.44		
		(D) Vd− and Vc−	7.47 ± 0.22		
	Biological effects	(E) vairimorpha only (C‐D)		0.61 ± 0.54	86.7%
		(F) varroa only (B‐D)		−0.15 ± 0.27	30.6%
		(G) vairimorpha + varroa (A‐D)		0.81 ± 0.27	98.9%
	Interaction	Effect of vairimorpha in the presence of varroa (G‐F)		0.96 ± 0.37	99.4%
		Effect of varroa in the presence of vairimorpha (G‐E)		0.20 ± 0.64	62.1%

*Note:* Viral load predictions are shown for the four combinations involving varroa‐positive (Vd+) and varroa‐negative (Vd−) island status and vairimorpha‐positive (Vc+) infection status: (A) Vd+/Vc+, (B) Vd+/Vc−, (C) Vd−/Vc+, and (D) Vd−/Vc−. Biological effects are expressed as log_10_ effect sizes calculated as the mean expected difference in log10‐load estimates between different co‐infection scenarios (e.g., vairimorpha only = C (6.89 ± 0.25)—D (6.62 ± 0.15)). Interaction terms quantify the additional effect of one parasite/pathogen in the presence of the other. Pr (> 0) indicates the posterior probability that the estimated effect size is positive (i.e., increases viral load).

## 4 Discussion

The origin of *A. mellifera* in the Azores dates back to the 16th century, when Portuguese settlers introduced the first colonies during the human colonisation of the archipelago. In the absence of native honey bees, these introductions were essential for providing honey and wax to the inhabitants. Genetic analyses suggest that the founding stock originated in northern mainland Portugal and that present‐day Azorean populations are, therefore, derived from *A. m. iberiensis* (lineage M), native to the Iberian Peninsula (Ferreira et al. 2020).

More recently, during the second half of the 20th century, additional, legal and illegal, introductions of commercial stocks belonging to the divergent honey bee lineage C have not only reshaped the genetic composition of Azorean populations (Henriques et al. 2025), but also altered their parasite and pathogen landscapes (Lopes et al. 2022; Lopes et al. 2024a; Lopes et al. 2024b). In particular, these movements led to the introduction of the varroa mite and its associated pathogens, first on Pico in 2000, then on Flores in 2001, and lastly on Faial in 2008 (Ferreira et al. 2020). To prevent further spread of the varroa mite within the archipelago, honey bee movements from the three infested islands, as well as new imports, are now prohibited under EU regulations recognising the Azores as a varroa‐free territory (Commission, E 2019), a status that is maintained through continuous official surveillance programmes, including regular apiary inspections and active screening for varroa. In contrast to varroa, whose introduction was readily detected by veterinary authorities, the timing and route of arrival of *V. ceranae* in the Azores remain unknown (Lopes et al. 2022). Given the resulting complex parasite and pathogen landscapes, shaped by multiple introduction events and varying across islands, understanding pathogen‐parasite interactions within these distinct epidemiological contexts and isolated environments becomes particularly important.

We showed that vairimorpha infection was associated with increased loads of BQCV and LSV, further supporting the claim that these pathogens interact synergistically (Bailey et al. 1983; Runckel et al. 2011; Daughenbaugh et al. 2015; Doublet, Labarussias, et al. 2015; Traynor et al. 2016; Gajda et al. 2021). By screening for multiple LSV strains, we could also show that this interaction was evident for LSV‐2, but not for LSV‐3 or the newly reported LSV‐9 (Lopes et al. 2024a). Remarkably, the effect of vairimorpha on BQCV and LSV‐2 loads was stronger on the varroa‐infested islands than on the varroa‐free islands, suggesting that the presence of the mite may further modulate vairimorpha‐virus interactions. Using a more complex model that simultaneously incorporates both biotic stressors, our results further underscore the role of varroa in amplifying the effect of vairimorpha on viral loads, particularly in the case of LSV.

In contrast to BQCV and LSV, it is less clear whether or not vairimorpha impacts the development of DWV infections. Some studies reported correlational evidence for a synergistic interaction between vairimorpha and DWV (Zheng et al. 2015; Little et al. 2016; Bordin et al. 2022), while others suggested an antagonistic effect (Costa et al. 2011; Doublet, Natsopoulou, et al. 2015) or even no interaction (Martin et al. 2013). In the Azores however, no significant additive effect, either positive or negative, was observed between vairimorpha and DWV irrespective of the strain of DWV involved or the varroa status of the islands (Table 2). No assessment could be made for DWV on the varroa‐free islands Santa Maria, São Miguel, and Graciosa, since these islands were coincidentally also practically free of vairimorpha (Figure 2). The conclusions from the varroa‐infested islands were much more robust, due to the higher prevalence of both vairimorpha and DWV on these islands, particularly on Pico (Figure 1), with both DWV‐A and DWV‐B dominant colonies generously represented among Vc + and Vc‐ colonies, generating a greater volume of usable data. The higher prevalence and abundance of DWV, together with the dominance of DWV‐A and DWV‐B on varroa‐infested islands, likely reflect the high efficiency with which these strains are transmitted by the mite (Yañez et al. 2020). DWV‐C appears able to persist in the absence of varroa but is displaced by DWV‐A and DWV‐B under varroa pressure (Lopes et al. 2024b). This shift may reflect differences in transmission dynamics, where DWV‐A is primarily transmitted mechanically but with reasonable persistence inside the mite (Posada‐Florez et al. 2019) whereas DWV‐B has also a well‐developed ability to replicate and accumulate inside the mite, enhancing vector competence and facilitating more efficient transmission (Ryabov et al. 2022). These strain‑specific adaptations to varroa transmission give DWV‑A and DWV‑B a competitive advantage over other, presumably less‐well adapted strains of DWV (e.g., DWV‐C, DWV‐D; de Miranda et al. (2022)) in varroa‑infested environments, but not necessarily in varroa‐free environments, making the varroa‐free islands a potential refuge for these minor strains (Lopes et al. 2024b).

SBV was only present on varroa‐infested islands with higher loads in Vc‐ colonies. SBV is a brood disease, transmitted by adult worker honey bees through glandular secretions (Yañez et al. 2020). SBV has little effect on adult honey bees ( *A. mellifera* ) except for a reduced affinity for pollen consumption or foraging, resulting in accelerated ageing and differential nectar foraging (Bailey and Fernando 1972). Since vairimorpha infection intensity is stimulated by pollen consumption (Jack et al. 2016), there is a behavioural‐physiological basis for a negative relationship between vairimorpha and SBV prevalence in adult bees.

The mechanisms underlying the synergistic interactions between vairimorpha, BQCV, and LSV are unclear, especially in relation to the apparent strain‐specificity of the vairimorpha‐LSV interaction. Vairimorpha, BQCV, and LSV all infect the honey bee midgut (Daughenbaugh et al. 2015; Higes et al. 2020). Vairimorpha could therefore facilitate BQCV and LSV infiltration into the honey bee tissues and haemolymph through the lesions it causes in the cells lining the midgut (Martín‐Hernández et al. 2018; Al Naggar and Paxton 2020). Vairimorpha also reduces honey bee immunocompetence (Antúnez et al. 2009; Higes et al. 2013), thereby facilitating the opportunistic development of BQCV and LSV infections. These mechanisms are not mutually exclusive, and it may well be that both are similarly relevant, with both types of pathogens facilitating each other's infections in a synergistic manner. What is more difficult to explain is the differential response of LSV strains to colony‐level vairimorpha infection, with only LSV‐2 showing a strong association with vairimorpha presence, and how this differential response is also affected by the presence of varroa, with the strength of the vairimorpha/LSV‐2 relationship stronger on the varroa‐infested islands than on the varroa‐free islands. Interestingly, while the effects of vairimorpha and varroa on BQCV are more balanced, yet still combine to elevate viral loads beyond the effect of either stressor alone, the pattern for LSV is distinct. In agreement with previous findings (Lopes et al. 2024a), varroa alone did not affect LSV viral loads, but its presence significantly amplified the effect of vairimorpha, with a high probability. Together, these results suggest that vairimorpha–virus interactions are not uniform across viral taxa and that varroa can reshape these interactions in virus‐specific ways that are largely unknown.

The answers to these questions are beyond the scope of this study, where the long‐term isolation of the honey bee populations (and their pathogens) on the different islands has contributed strongly to the independent epidemiological and evolutionary trajectories of their pathogens (Lopes et al. 2024a, 2024b). This would have introduced a considerable stochastic element to the core nature of the data, particularly between the islands (i.e., the varroa effect), which were not possible to be fully accounted for in the statistical analyses, despite the considerable number of colonies included. These questions ideally require closely controlled co‐infection experiments aimed at identifying the mechanisms of synergy between varroa, vairimorpha and the various viruses and their strains.

In conclusion, the unique setting and long‐term geographic isolation of the bee populations of the Azores island archipelago led to the independent parallel development of unique combinations of parasites and pathogens. This, in turn, enabled the identification of specific synergistic interactions between vairimorpha and several honey bee viruses, especially BQCV and LSV‐2, as well as how the long‐term presence of varroa strengthens these synergies. By unravelling these complex interactions, we gain deeper insights into the multifaceted threats faced by honey bee colonies.

## Funding

This work was supported by Portuguese funds through Foundation for Science and Technology (FCT, Portugal) and by FEDER (Fundo Europeu de Desenvolvimento Regional) through the program COMPETE 2020‐POCI (Programa Operacional para a Competividade e Internacionalização) in the framework of the project BEEHAPPY (POCI‐01‐0145‐FEDER‐029871). This work was supported by national funds through FCT/MCTES (PIDDAC): CIMO UID/00690/2025 (10.54499/UID/00690/2025) and UID/PRR/00690/2025 (10.54499/UID/PRR/00690/2025); SusTEC, LA/P/0007/2020 (DOI: 10.54499/LA/P/0007/2020). ML was funded by the Swedish Research Council (2017‐03963). JdM was partially funded by grant 2022‐01462 from the Swedish Research Council for Sustainable Development (FORMAS).

## Conflicts of Interest

The authors declare no conflicts of interest.

## Supporting information


**Table S1:** List of the colony IDs, collection dates, locality IDs and Azorean island from which the honey bee samples in this project were collected.
**Table S2:** Distribution of the honey bee samples by island and collection year.
**Table S3:** Comparison of three alternative Priors distributions (*μ*_*α*_~*N* (0,100), ~N (15,100) and ~*N* (3,2)) on the virus load estimate, effect size and the Bayesian probability of this effect size for the effect of the presence of varroa and/or vairimorpha, plus their interaction, on the presence and amount of five common bee viruses (BQCV, DWV, SBV, LSV and CBPV) in honey bee colonies on the Azores island archipelago during 2014, 2015 and 2020, as estimated through Observation Model 2. Probabilities > 95% indicate a strong effect, > 90% a moderate effect, > 70% weak effect and < 70% no effect.

## Data Availability

The dataset used in the study and the R scripts for Bayesian analysis are available in the dados.ipb.pt repository under the following identification: https://doi.org/10.34620/dadosipb/OPBXJE
